# Antenna Integration for Millimeter-Wave RF Sensing and Millimeter-Wave Communication Mountable on a Platform

**DOI:** 10.3390/s24154838

**Published:** 2024-07-25

**Authors:** Jaewon Koh, Hongsik Park, Woogon Kim, Seongbu Seo, Yejune Seo, Sungtek Kahng

**Affiliations:** 1 Department of Information & Telecommunication Engineering, Incheon National University, Incheon 22012, Republic of Korea; rhwodnjs91@inu.ac.kr (J.K.); p0306ok@inu.ac.kr (H.P.); wgon1002@inu.ac.kr (W.K.); castlerich@inu.ac.kr (S.S.); 2 NS-Satellite RTDC ITRCenter, Incheon National University, Incheon 22012, Republic of Korea; m.june@inu.ac.kr

**Keywords:** array antenna, millimeter-wave, signal strength, radio sensor, reflecting surface, transmission and reflection

## Abstract

An array antenna for millimeter-wave communication and an array antenna for millimeter-wave sensing are designed and put together into one structure. Because millimeter-wave signals become weaker fast with the increasing distance and any kind of error in the required functions of the antenna has to be minimized, pointing error from the target direction should be prevented. The device is a millimeter-wave sensing antenna with high directivity to check the straight link between the TX and RX sides of wireless communication. A 24 GHz 8-by-16 array antenna which generates stronger signals for sensing resolves the drawback of a 28 GHz 1-by-4 array antenna that is commonly seen in 5G wireless terminals. The sensing and communication antennas are integrated as a planar structure mountable on platforms, which is investigated with regard to forming wireless links over a distance of several meters with an input power of less than 0 dBm. Additionally, in the event of a reflecting surface disturbing the straight path and worsening the pointing error in RF signal transfer, the dual-capability of the combination is presented on the basis of intuitive electromagnetic experiments.

## 1. Introduction

Wireless communication has been developed and accepted in industries building the infrastructure of cities and in profit-making areas. For example, it makes cellular networks possible on a regional scale and allows satellite links to happen on a global scale. From its first generation to the present day, it has been evaluated to meet the industrial standard or public demands, and it has been tailored to comply with certain requirements, such as connectivity to nodes of the network and high data transmission rates. These are being realized and put into more use in the 5G mobile service era through larger bandwidths and low-latency millimeter-wave (henceforth, mmW) wireless systems [1,2]. Even in the 5G era’s infancy, scholars have begun talking about 6G, which might confuse people with presumption that 5G has been skipped. However, the two paradigms have a lot of expectations in common. In accordance with white papers and books on standards that 3GPP and mobile carriers have published, software and hardware for the 5G are to be also used for the 6G. What differentiates 6G from 5G, aside from the fusion of separate functions, are the three-dimensional communication topologies interconnecting the ground networks with the wireless signal flows to satellites. The vertical paths will be accessed by the horizontal networks that are conventionally limited in expanding the coverage and raising the speed, but the 3D network diagram is drawn and schematized between the caller and receiver in order to find possible routes and estimate the costs, and to make the suggestion that an optimized route leads to a cost-saving solution of fast data exchange. While this non-terrestrial networking (NTN) is being studied and worked on very actively, the fusion of separate functions is examined in terms of its merits and hurdles as another feature of 6G. 

The fusion of technologies from different fields is exemplified by sensing and communication. This is so called integrated sensing and communication or ISAC. Not only 6G but also other communication sectors, like satellite links, have combined one technology with another. The SAR satellite is equipped with communication payload-neighboring RF sensors to collect the data of reflected waves from the Earth. Primarily, as more and more functions and their hardware blocks are demanded for wireless communication, and the size of the wireless terminal is limited as a result of users’ preference, integration of different functions is required. Secondarily, shortcomings of two functional blocks can be improved by mixing them after they are juxtaposed to find points to compensate for. A sensor can use the mmW communication channel to tell its status to the controller. The beamforming antenna of the communication system takes the information on the direction of the infra or nomad in the IoT network that a sensor generates. The information is not polluted much by noise because the frequency of the sensor differs from that of the communication device. ISAC is introduced with a great number of use-cases. One of them is a vehicle having the array antenna as the communication device and autonomous radar as the sensor. Another is the LEO satellite, which is connected to the ground by the communication channel and refers to the location data received by the sensor from the surface. It is worth checking what is presented in the selected literature and reports on the ISAC and its antenna technologies.

ISAC can be depicted in various ways but is embodied from the viewpoint of waves from antennas mounted on vehicles to allow dual-capability as follows. 

**Figure 1:**
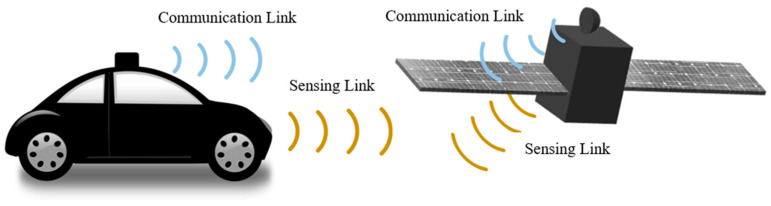
The sensing and communication functions are integrated into the surface of the moving vehicle, like a car or satellite.

Even if ISAC has been recently coined, there have been techniques to place sensing and communication devices together on a platform, as shown in Figure 1. Whether separated or integrated, modern automobiles are expected to carry out functions of wireless communication under GSM, WiFi, and LTE-A protocols and RF sensing for collision avoidance. As for the car, it is linked with the infrastructure of mobile communication, such as a base-station, which is peeped into by the automatic updating of the map in the navigation module. This is made possible by the antenna on the top of the car. A radar used as the sensor is put at the front of the car to play a role in detecting the presence of an object ahead. Different frequencies are allotted to the communication link and sensing link. Similar to that, a satellite uses the aforementioned channels, allowing the platform to connect to the ground station and gather the information on water, fields, forests and mountains transformed from the reflected waves. As with the SAR satellite, RF sensing, though it is utilized for imagery, counts on the communication channel to maintain its quality and to adjust the positioning of the aperture. These applications share basic principles with the scenarios and objectives of the articles examined as flows. Gao et al. add massive MIMO to ISAC and apply compressed sampling to the new approach [3]. Information on the state of the high-dimensional channel comes with a reduced overhead, and the quality of radar imaging improves. Instead of using the MIMO, beamforming can be brought to ISAC, as shown in M. A. Islam and J. Wang’s work [4,5]. The former analyzes the function of in-band full-duplexing being combined with hybrid beamforming to detect the direction of the incoming signal. The latter models the system where the downlinks from the tower to multiple vehicles are backed by multiple beams. Similar to [5], Zhuo et al. consider the multi-beam in ISAC, but they assume uncorrelated beams and calculate the channel capacity [6]. Joung et al. apply zero-forcing beamforming and power allocation to their analysis on inter-user and inter-ISAC antenna interference [7]. The baseline work is changed to sparse vector coding in Zhang et al., but the operation of communications is improved by lowering side-lobes of the beam [8]. ISAC is relatively a young area and is mostly approached and understood with communication theories. Some of the articles on ISAC deal with hardware development and experiments. Lu et al. built a 2.4 GHz radar connected to the same frequency wireless module [9]. An impedance matching circuit for the duplexing function takes the feeding part of a microstrip circular patch, as in [10]. Temiz et al. operated a radar at 2.4 GHz as an RF sensor assisting an ISM-band wireless link [11]. Away from the low frequency and using the mmW band, Huang et al. designed a G-band receiver for enormously wide-band-based high data rates [12]. The receiver adopts a waveguide horn antenna. However, inexpensive and mmW solutions are needed.

In this paper, an antenna module less costly and more efficient in physical implementation is designed for integrated RF sensing and communication, with both in mmW bands. As illustrated below, the sensing and communication use different frequencies.

**Figure 2:**
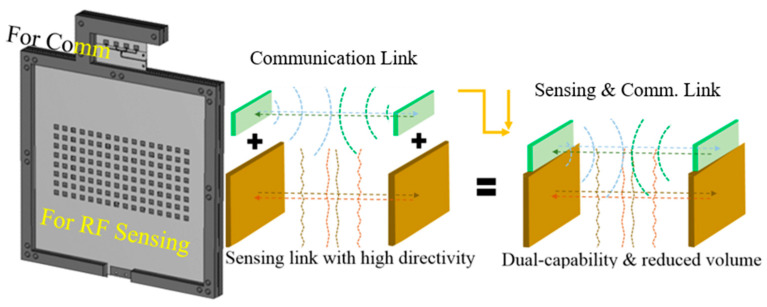
The proposed module of sensing and communication both in mmW-bands.

Figure 2 is the platform where the communication and RF sensing antenna blocks are integrated. The communication should work at 28 GHz, aimed at 5G and satellite-linked 6G. Meanwhile, the RF sensing antenna has to be operated at 24 GHz for use in in radar systems for automobiles and UAMs. The antennas take after the structures presented in [13]. However, differently from them, the antenna for the mmW communication terminal here is combined with the mmW radar antenna by placing feed-lines perpendicular from one bloc to the other in case of proximity interference, in order to check the direct path from the transmitter (TX) to the receiver (RX), which mitigates the pointing error. After assembling the two blocks, experiments are conducted to examine the signal strengths of the sensor and communication links over distances of many wavelengths with LOS and reflecting objects in terms of electromagnetic propagation.

## 2. Design of the Antenna for RF Sensing and That for Communication

Prior to the stage of the aforementioned assembly, the elemental antennas for the proposed module are designed. The antennas for the two blocks are required to work at mmW bands, which are appropriate for fast data transmission in communication and a very pointy beam in sensing. Both of the antennas take the forms of the array antenna to create narrow beams, and the physical sizes can be small due to the shorter wavelengths of the K and Ka bands. First, the antenna for communication is presented. 

**Figure 3:**
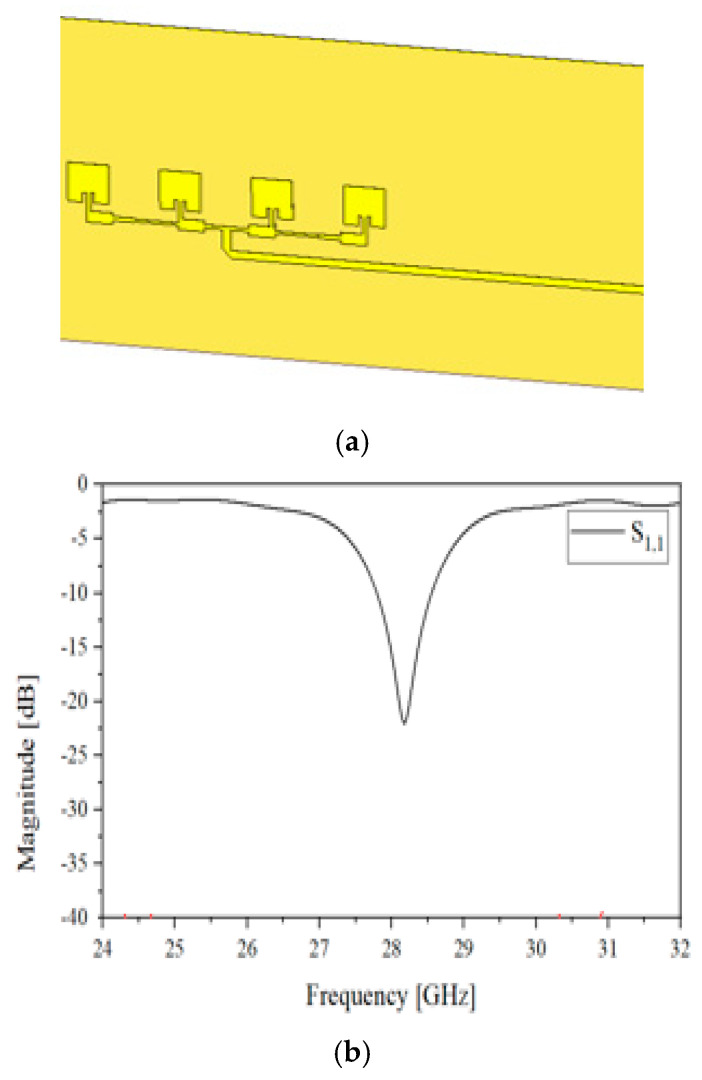

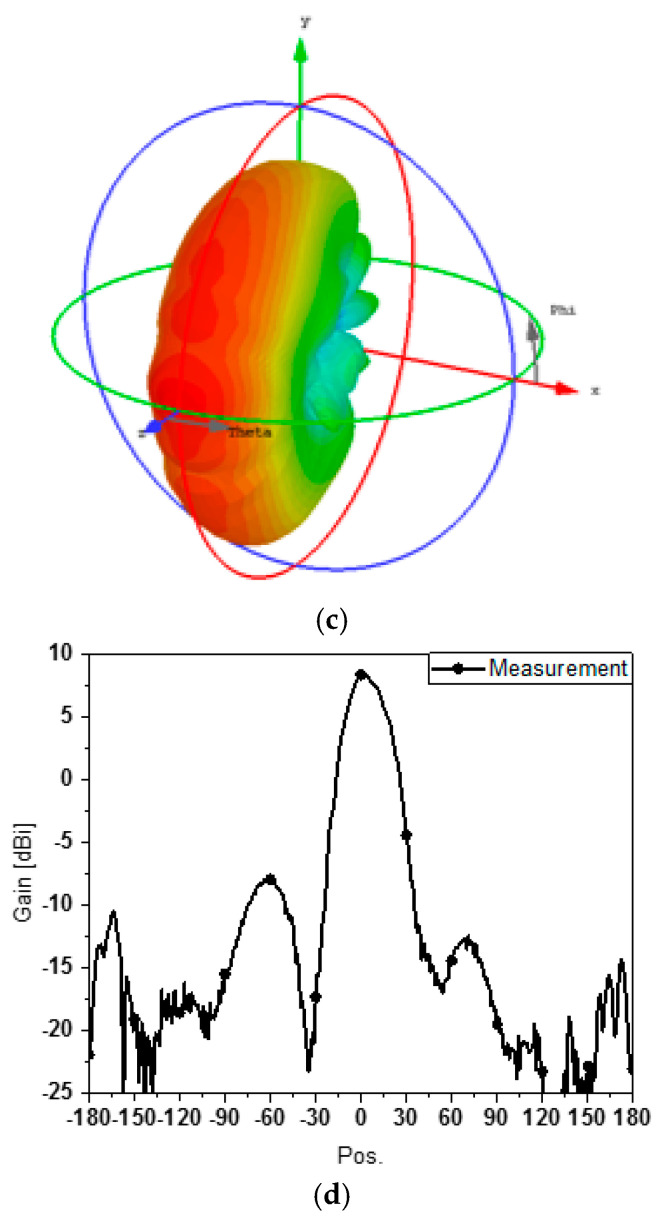
The antenna for mmW communication. (**a**) Geometry; (**b**) S_11_ as the reflection coefficient of the antenna; (**c**) far-field pattern of the one-dimensional antenna expressed in from red (strongest) through green (middle) to blue (weakest); (**d**) measured beam-pattern.

Presented in Figure 3a, it is a one-by-four array antenna consisting of the rectangular microstrip patches and the power divider. The patch is 2.8 mm long, the 1 × 4 array stretches 21.5 mm, and the substrate is 46.2 mm × 23.1 mm wide. The thickness of the metal pattern is 1 oz. The feed is horizontal to avoid EM noise from the patches and other block. Note that it is not difficult to have a larger array, but the one-by-four array is commonly seen in the layout of the commercial 5G mobile handset and adopted in the access point equipment. This structure is made to have good impedance matching at 28 GHz, as this is the target frequency’s so-called FR2 band. S_11_ is less than −10 dB, as in Figure 3b. This results in the far-field pattern as shown in Figure 3c,d. The beam pattern is also known as the fan beam. This is quite typical of the one-dimensional array with the resonance at this high frequency. Second, the antenna for the RF sensing block is constructed to be suitable for high directivity, as can be easily found from practices in the development of radar modules. 

**Figure 4:**
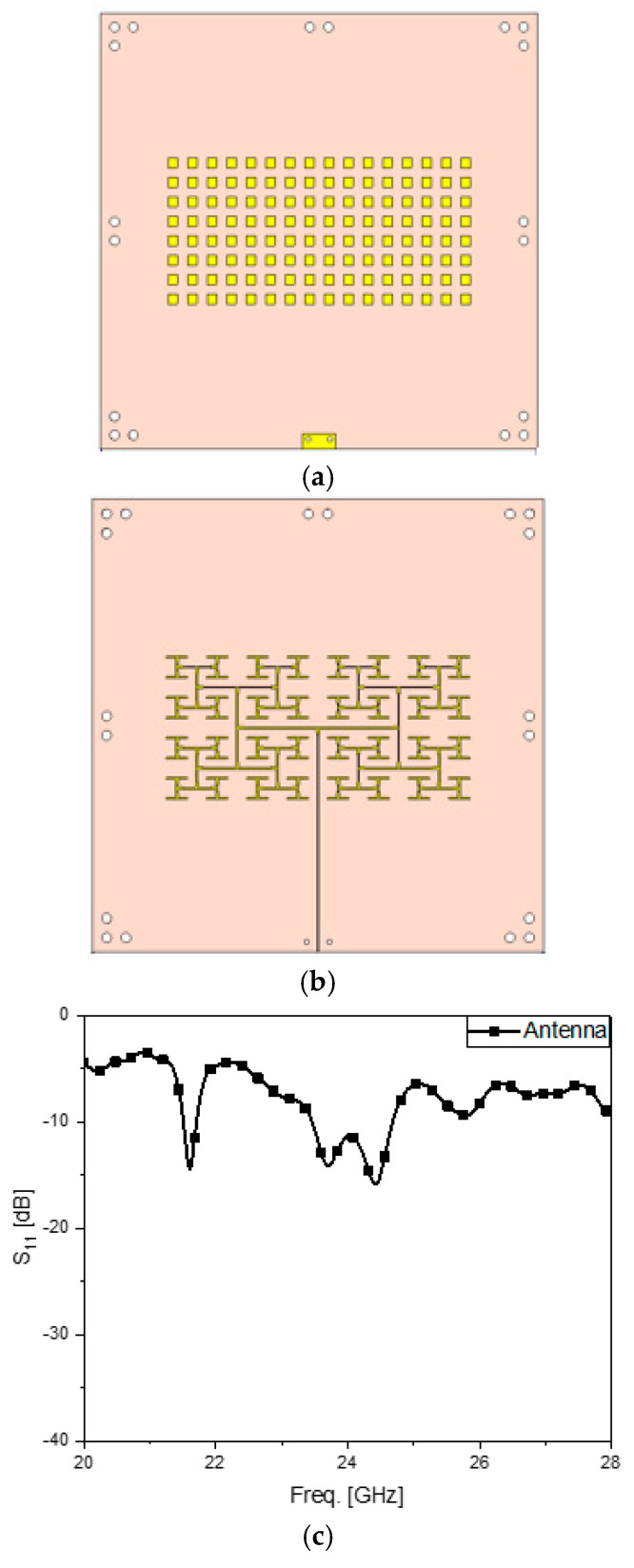

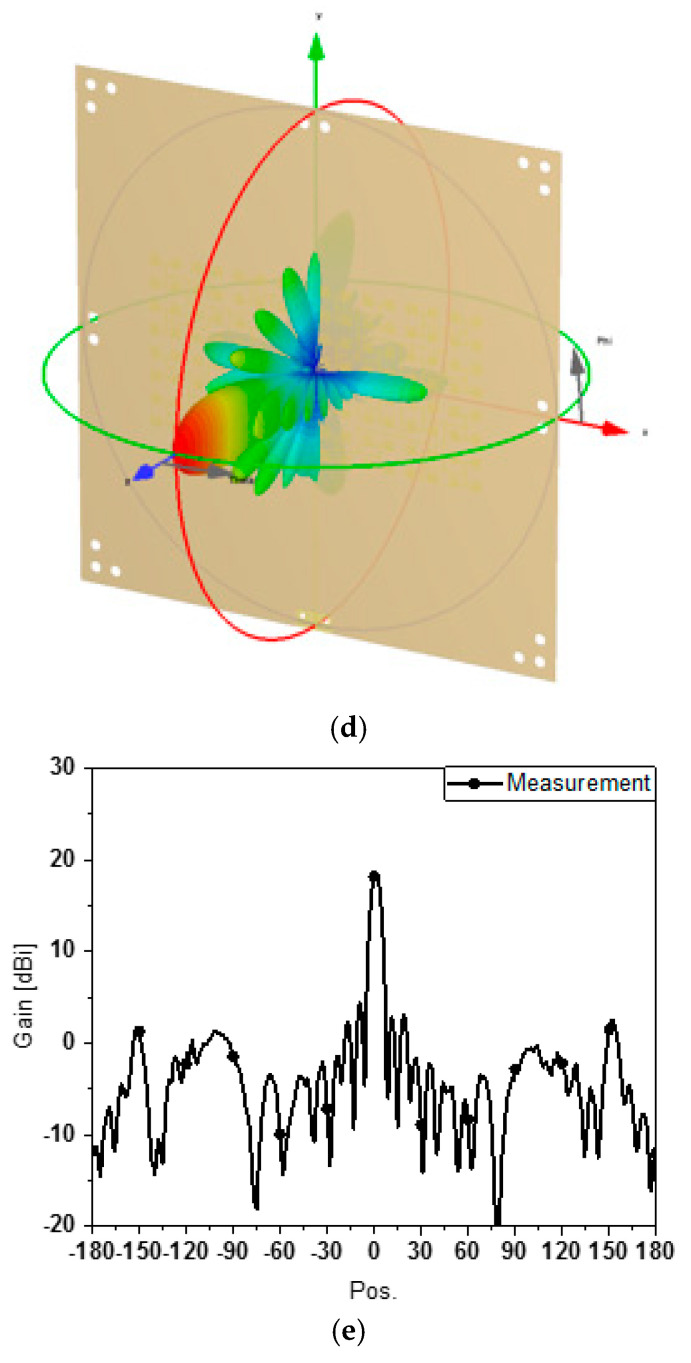
The antenna for RF sensing. (**a**) Front side of the array. (**b**) Backside of the array. (**c**) S_11_ of the antenna (**d**). Far-field pattern of the two-dimensional array antenna expressed in from red (strongest) through green (middle) to blue (weakest). (**e**) Measured beam-pattern.

Figure 4 elaborates on the antenna for the RF sensing block. This two-dimensional structure is an 8-by-16 array. The area of the metal pattern on the front side of the antenna is 4.7 cm × 9.6 cm, and this is based on the substrate, the area of which is equal to 14 cm × 14 cm. RT4350B is adopted as the dielectric substrate to hold 128 0.3 cm × 0.3 cm-sized radiating patches. In order to excite the antenna, a complicated-looking power divider that has branches with as many as 128 patches is designed and placed as the metal pattern on the other side of the substrate. A feed-line, laid vertically to avoid the interfering fields from the other block, is centered at the branches. The radiating elements are connected to the ends of the branches through metal vias penetrating the metallic ground between the front side, as in Figure 4a, and the back side, as in Figure 4b, of the structure. The design is validated by investigating S_11_ as the reflection coefficient at the input port of the power divider, as well as the radiation performance. If it were a chip-based active beamforming antenna, like AiP, because each of the patches is directly fed by its designated pin of the chip, the designer does not need a power divider. Nonetheless, as the passive array antenna, there is one port that introduces the RF signal as the input power through large branches to the small branches where equally divided power is desired. When the frequency of interest is low, even if there are many branches needed for a power divider, the design is not complicated. However, when the operating frequency must be as high as the mmW bands, it becomes much harder to design a power divider with a great number of branches, since the electromagnetic wave in the component becomes attenuated faster than at low frequencies, and the power division ratios at the end branches tend to be unbalanced and unequal. This phenomenon deteriorates the impedance matching of the input port. To solve these problems, the power divider here has been designed well, which is proven by the S_11_-curve in Figure 4c. Using 24 GHz as the target frequency, S_11_ is below −10 dB in the frequency band. From the one and only input port to the many branches and radiating patches in the power-dividing circuit, it is apparent that the impedance is matched. The RF signal divided through the feeding circuit reaches the rectangular patches and makes them radiate the electromagnetic fields to the free-space. This can be verified by the far-field pattern. Figure 4d,e has the three-dimensional beam-pattern plotted just over the front side of the structure for the sake of convenience. The far-field pattern has a narrow beam resulting in high directivity that is adequate for building a wireless link with RF sensors, such as radar and microwave imaging apparatus. When there is an object to be detected and it is located very far away from the source of radiation, a highly directive beam makes the returning signal stronger than other cases. 

## 3. Integrating the Antennas for RF Sensing and Communication and Experiments

The elemental antennas which were designed and explained in the previous section are integrated into one module to be the core part of the ISAC equipment shown below. The communication block is located at the top of the surface-sensor block because the antenna for communication, which works at the higher frequency, is the smaller of the two antennas. 

**Figure 5:**
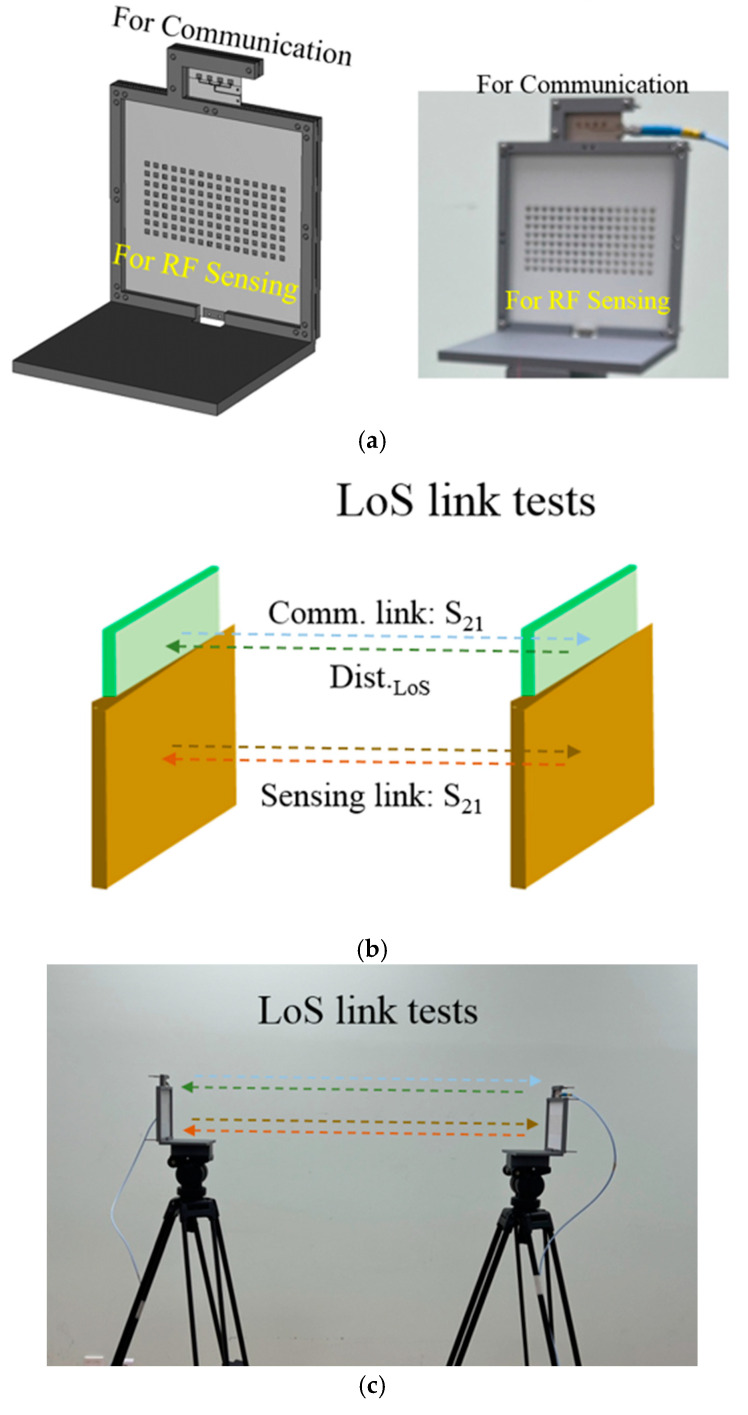

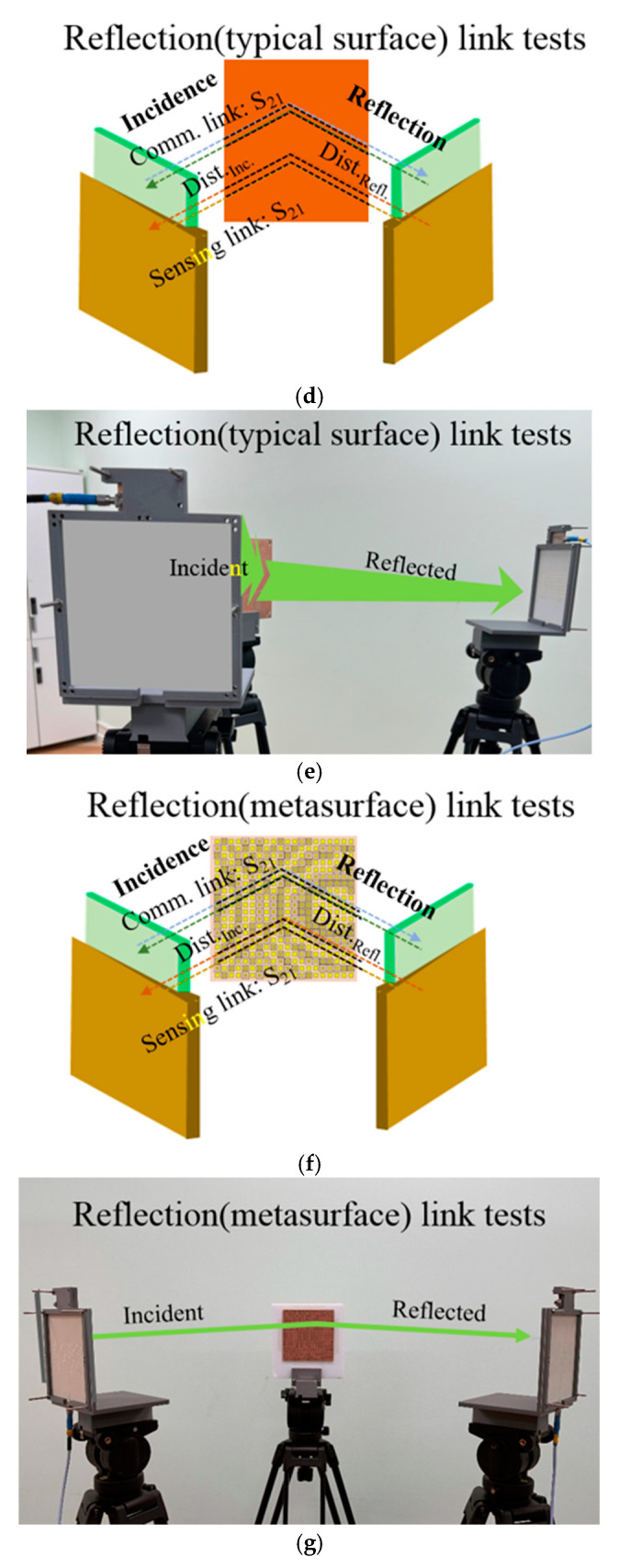

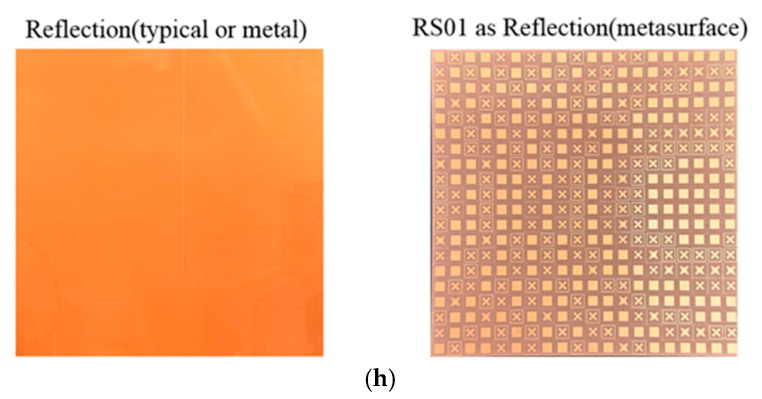
The module integrating the communication and surface-sensing antennas put into the experiments. (**a**) Prototype of the module. (**b**) Scheme of LoS link tests. (**c**) Real LoS link tests. (**d**) Scheme of the typical reflection link tests (**e**). Scheme of the typical reflection link tests. (**f**) Scheme of RS01(metasurface) reflection link tests. (**g**) Scheme of RS01(metasurface) reflection link tests. (**h**) Typical metal reflector and a 28 GHz-beam-tilting metasurface.

Figure 5 displays the scenarios and experimental configurations used to examine the functions and properties of the small and different surface conditions, like in Figure 5a. The communication and sensing links on the line-of-sight (LoS) are initially observed as in Figure 5b, which is realized by Figure 5c. Secondly, the direct path is changed to the sum of the incident and reflected paths by assuming an object. This mimics the scattering of the wireless signal caused by reflections from cars or buildings in an urban site. As mentioned earlier, the pointing error of the radiated wave seriously influences the mmW system, and it is assisted by the information acquired by the sensor which recognizes a barrier in the communication link. This is one of the reasons why ISAC is introduced to ensure good quality in 6G mobile services. The EM links of the ISAC module are tested with reference to reflection by the typical conducting plane as in Figure 5d,e, and with a reflecting metasurface, as in Figure 5f,g as a non-conventional object to be sensed. Its characteristics are mentioned in Appendix A. The two reflecting surfaces with the area of 11.5 cm × 11.5 cm are compared in Figure 5h. The distance between the two ports of the LoS tests is denoted as *Dist._LoS_* in Figure 5b, and there is nothing in the path between the two sides, as in Figure 5c. The straight path becomes *Dist._RoundT_*, broken into *Dist._Inc._* as the distance of the incident segment and *Dist._Refl_* as the distance of the reflected segment, as shown in Figure 5d,f. The frequencies dealt with here belong to a very high frequency group, in other words, 28 GHz and 24 GHz, and most of surfaces of the structures may seem like conducting facets to the impinging mmW signals. The left picture in Figure 5h is a copper plane. These days, reconfigurable intelligent surfaces (RISs) draw attention from researchers studying 6G mobile service as a way of reducing the blockage error, which is rooted in the reflective array as the metasurface [14]. As such, it also hinders the signal.

**Figure 6:**
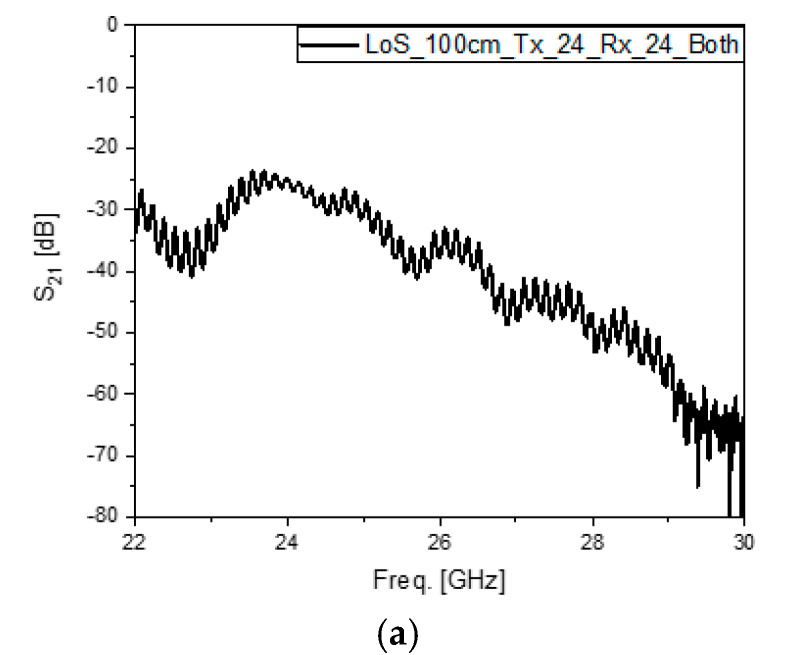

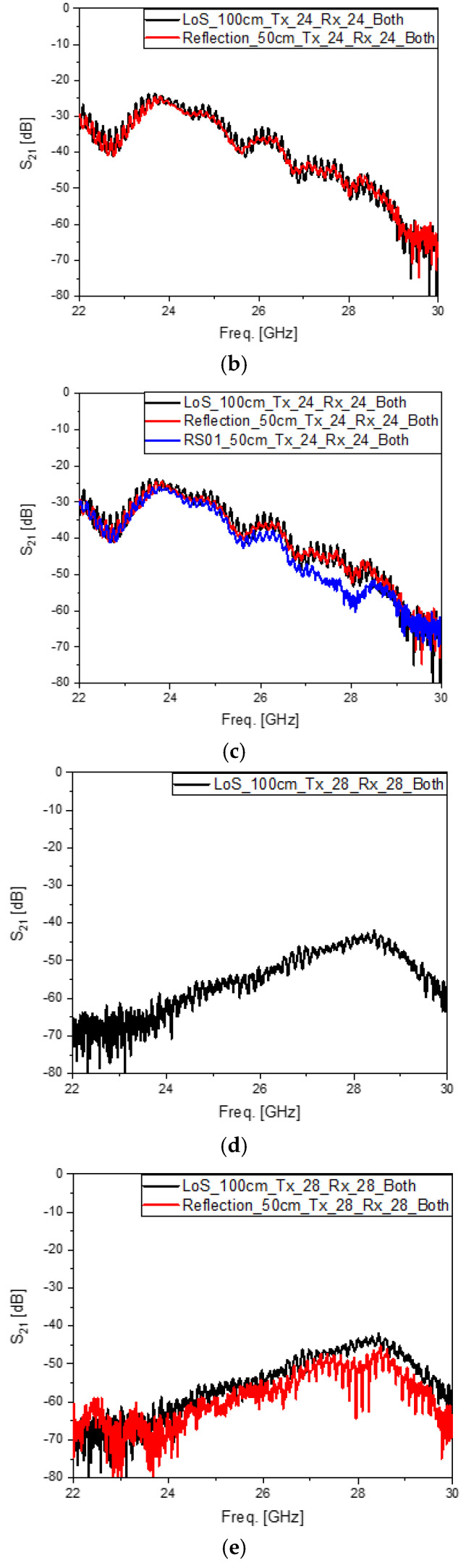

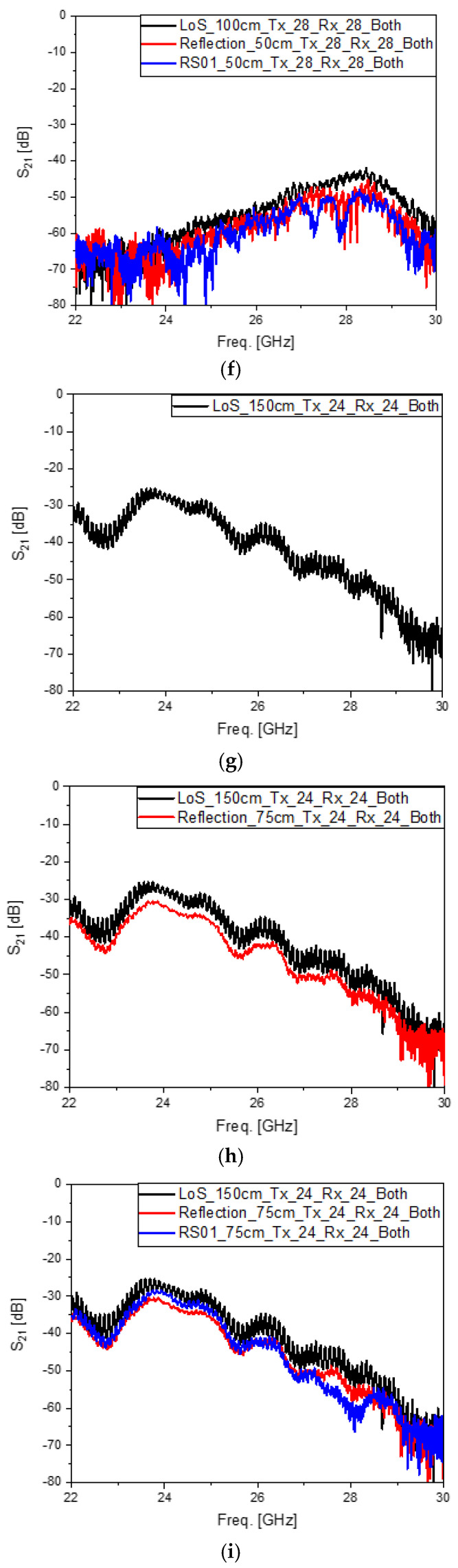

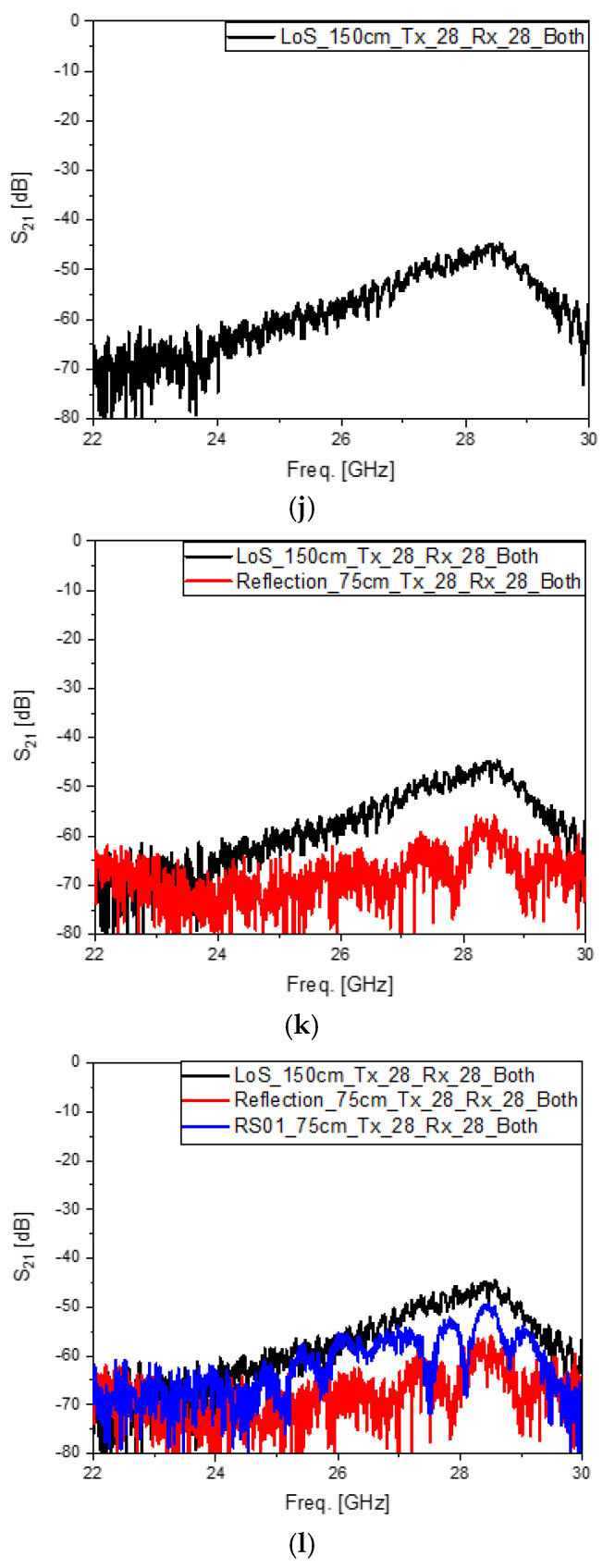
The results of testing various links between the TX and RX as the integrated module of the communication and surface-sensing antennas by observing S_21_ as the transfer coefficient. (**a**) Sensing signal LoS of *Dist_LoS_* = 10^3^ mm. (**b**) Sensing signal reflection by the metal plane with *Dist_RoundT_* = 10^3^ mm vs. LoS. (**c**) Sensing signal reflection by RS01 as the metasurface with *Dist_RoundT_* = 10^3^ mm vs. reflection by the metal plane vs. LoS. (**d**) Comm. signal LoS of *Dist_LoS_* = 10^3^ mm. (**e**) Comm. signal reflection by the metal plane with *Dist_RoundT_* = 10^3^ mm vs. LoS. (**f**) Comm. signal reflection by RS01 as the metasurface with *Dist_RoundT_* = 10^3^ mm vs. reflection by the metal plane vs. LoS. (**g**) Sensing signal LoS of *Dist_LoS_* = 1.5 × 10^3^ mm. (**h**) Sensing signal reflection by the metal plane with *Dist_RoundT_* = 1.5 × 10^3^ mm vs. LoS. (**i**) Sensing signal reflection by RS01 as the metasurface with *Dist_RoundT_* = 1.5 × 10^3^ mm vs. reflection by the metal plane vs. LoS. (**j**) Comm. signal LoS of *Dist_LoS_* = 1.5 × 10^3^ mm. (**k**) Comm. signal reflection by the metal plane with *Dist_RoundT_* = 1.5 × 10^3^ mm) vs. LoS. (**l**) Comm. signal reflection by RS01 as the metasurface with *Dist_RoundT_* =1.5 × 10^3^ mm vs. reflection by the metal plane vs. LoS.

The experiments are conducted largely from four standpoints that are the sensing signal link vs. the comm. signal link, LoS vs. the round-trip link by reflection, the reflection of the typical conducting plane vs. the reflection by RS01, and a long distance vs. a longer distance. In detail and one by one, the first viewpoint means that the sensing signal link and comm. signal link are mainly driven by 24 GHz and 28 GHz signals, respectively. The second viewpoint is that the S_21_ values of the LoS and round trips by two different kinds of system are compared. The third point is that the strengths of the reflected signals by the copper plane and the metasurface are compared. The fourth viewpoint is that considering that the wavelengths at 24 GHz and 28 GHz are 12.5 mm and 10.7 mm, respectively, 1 m and 1.5 m are given as the distances of the direct and round-trip links, respectively. The input power of −3 dBm, which is a very low level of RF power, and the angles of incidence from TX and reflection to RX, which are set at 45°, are applied to all the tests here. Figure 6a–c shows the S_21_ of 24 GHz input signal cases with the distance *Dist_LoS._* and *Dist_RoundT_* = 1 m going with LoS, reflection by the metal plane vs. LoS, and the reflection by RS01 with the metasurface vs. reflection by the metal plane vs. LoS, in that order. Figure 6a,b presents what happens when a very highly directive beam for the direct path and the round trip of reflection by a large metal plane travels the same distance, i.e., their S_21_-curves are identical. This proves the electromagnetic theories. Compared with them, the S_21_ of the reflected signal due to RS01 behaves differently, which is inferred to be because non-conventional scattering occurs by the changed boundary condition of the reflecting surface [14,15,16,17,18,19,20]. Common to the three tests, the RX receives the strongest signal at 24 GHz, which was transmitted through the sensing channel. The next three cases focus on the communication link, which uses the 28 GHz signal. Figure 6d–f shows the S_21_ of the distance *Dist_Round_* and *Dist_RoundT_* = 1 m going with LoS, the reflection by the metal plane vs. LoS, and the reflection by RS01 of the metasurface vs. the reflection by the metal plane vs. LoS, respectively. Figure 6d,e shows a deviation of the curve of the round trip from that of the direct path, which results from a fraction of the fan beam from the 28 GHz 1D array going over the edge of the reflecting surface. *Dist_LoS_* = 1 m is already a very long distance equivalent to many wavelengths. Thinking of the low input power of the TX in the VNA, *Dist_LoS_* feels extremely far for mmW bands. Moreover, the distance is extended to *Dist_LoS_* and *Dist_RoundT_* = 1.5 m to confirm the advantage of the high directivity of the designed antennas. Figure 6g–i shows the S_21_ of 24 GHz input signal cases with the longer distance given to LoS, the reflection by the metal plane vs. LoS, and the reflection by RS01 of the Ka band reflect array vs. reflection by the metal plane vs. LoS, respectively. Figure 6g,h shows that even though they are very close, the error between them still occurs, since the new distance makes the finite sized plane look waning, and the returned signal becomes weaker than in Figure 6b. Despite there being a decrease in the signal strength, it is of a small scale because of the advantage of a very highly directive beam. Like in Figure 6c, the reflected signal from RS01, as in Figure 6i, has a different behavior from the other cases, which comes from the changed boundary condition surface. The last three cases relate to the communication link, which uses the 28 GHz signal and a distance of 1.5 × 10^3^ mm. Figure 6j–l shows the S_21_ curves of the LoS, the reflection by the metal plane vs. LoS, the reflection by RS01, and the the metasurface vs. the reflection by the metal plane, respectively. Figure 6j,k shows rather a large deviation of the curve of the round trip from that of the direct path, which occurs because a portion of the fan beam from the 28 GHz 1 × 4-array misses the reflecting surface, which is located at a farther position and looks shrunken as a result of the increased path. Interestingly, the returned 28 GHz signal is made stronger, reaching −50 dB instead −58 dB, when the metal plane is replaced by the metasurface, as shown in Figure 6l. This 8 dB jump indicates that when the reflecting surface is intentionally built for tilting the angle of reflection and gathering the electromagnetic flux more on to the reflection segment for either a sensing or communication link, the signal strength can be as high as the level of the LoS even when taking a detour, such as with the RIS. Two useful pieces of information are obtained in the following paragraphs. The first piece of information is related to the following mathematical expressions:

(1) $$P_{RF\_RX}{|}_{{Dist.}_{Ref}}=\zeta \times \frac{{{\left|E_{0}\right|}_{Freq.}}^{2}}{{{Dist.}_{Ref}}^{2}}$$

(2) $$P_{RF\_RX}{|}_{{Dist.}_{New}}=\zeta \times \frac{{{\left|E_{0}\right|}_{Freq.}}^{2}}{{{Dist.}_{New}}^{2}}$$

(3) $${\Delta P}_{RF\_RX}\left[\mathrm{dB}\right]=P_{RF\_RX}{|}_{{Dist.}_{New}}\left[\mathrm{dB}\right]-P_{RF\_RX}{|}_{{Dist.}_{Ref}}[\mathrm{dB}]=10{log.}_{10}\;\frac{{{Dist.}_{New}}^{2}}{{{Dist.}_{Ref}}^{2}}$$

where *P_RF_RX_*, ${{\left|E_{0}\right|}_{Freq.}}^{2}$, and ζ imply the RF power of the received signal expressed in S21, the initial value of the RF power, and the coefficient of the electromagnetic radiated power, respectively. The difference between the RX power levels from the LoS link with *Dist_Ref_* = 1 m and *Dist_New_* = 1.5 m is 3.5 dB according to the calculation. This is pretty much the same as the difference between the signal strengths. as the peaks of the S21 curves were observed to be −21.5 dB in Figure 6a and −25.1 dB in Figure 6g. The experiments comply with the theories, and it is possible to guess the change in the signal path length by watching the power level in this sort of measurement. The second piece of information is that though the sensing block and communication block are assembled as one planar stru1cture, the interference to other operating frequencies is very low, at −50 dB at 28 GHz for the 24 GHz radiation and −65 dB at 24 GHz for the 28 GHz radiation. This results from the separation of the frequencies even at the mmW band and due to the generation of high-directivity beam patterns from the physically small antennas.

## 4. Conclusions

As a primary work to build an ISAC system, the sensing and communication antennas have been designed and integrated into a module. With the elemental components of the ISAC module developed in this paper applied to 6G fast data transmission and resolution-improved RF sensors, 4-branch and 128-branch power dividers and 1X4 and 8X16 array antennas have been realized to work at 28 GHz and 24 GHz as mmW frequencies, respectively. The components show good impedance matching performance at the aforementioned frequencies by overcoming high and harsh attenuation along the transmission lines embedded in the ordinary substrate. The 1D array antenna generates a fan beam typically used for 5G and 6G terminals, and the 2D array antenna radiates a very pointy beam, as presented in the design procedure for sensing. The 8 dBi 28 GHz antenna is helped by the 19 dBi 24 GHz antenna in setting the direction of communication. After making the module with these elements, their functions were verified in various tests of the LoS, reflection by a metal plane and the reflection with RS01 as the metasurface in sensing mode, communication mode, and in terms of the change in the total distance. Because of the highly directive beams for the sensing and communication links, the tests with the distance of 1m to 1.5 m, considering wavelengths 12.5 × 10^−3^ m and 10.7 × 10^−3^ m, do not show rapid degradation. According to the experiments, when the straight path of the LoS is bent to the round-trip path by the conductor plane or the metasurface, the strength of the returned signal tends to decrease in a non-ignorable scale. However, the measurement of the received signal at 28 GHz reveals that if the boundary condition of the reflecting surface is engineered to prevent power loss, it can be improved, seeing as there was an increment of 8 dB compared to the typical metal plane. Additionally, low interference from one operating frequency to another has been verified as an advantage of the proposed design. Inter-frequency isolation is over 15 dB. This research is applicable to the initial work for realizing an mmW ISAC device where the fixed angles of the radiated waves from the antennas and a relatively large area, which are limitations of the present structure, are enhanced by using beamformer chips and optimized size reduction.

## Figures and Tables

**Figure A1:**
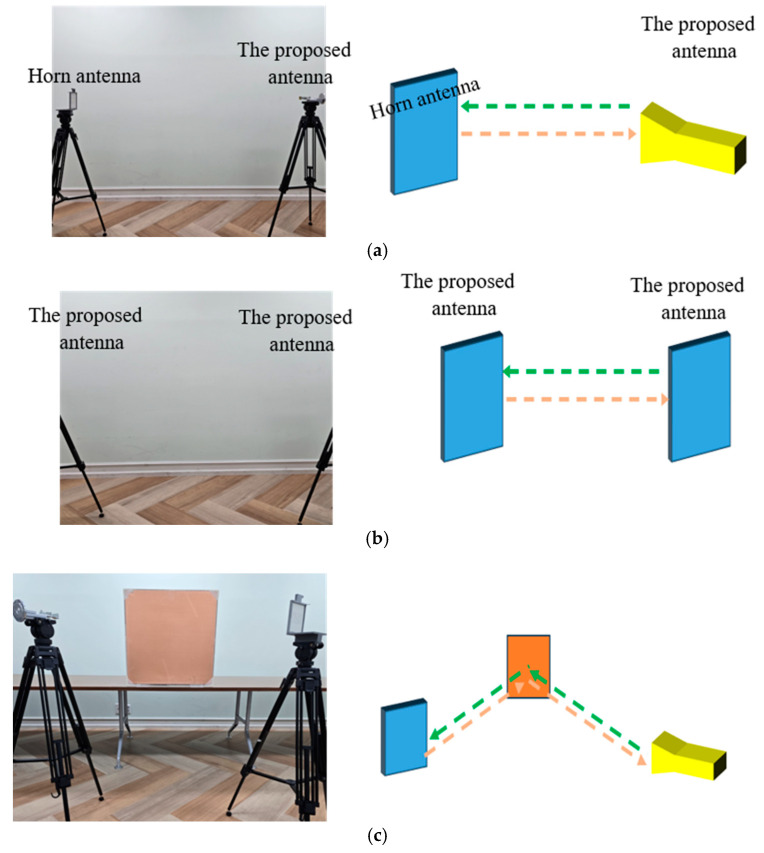

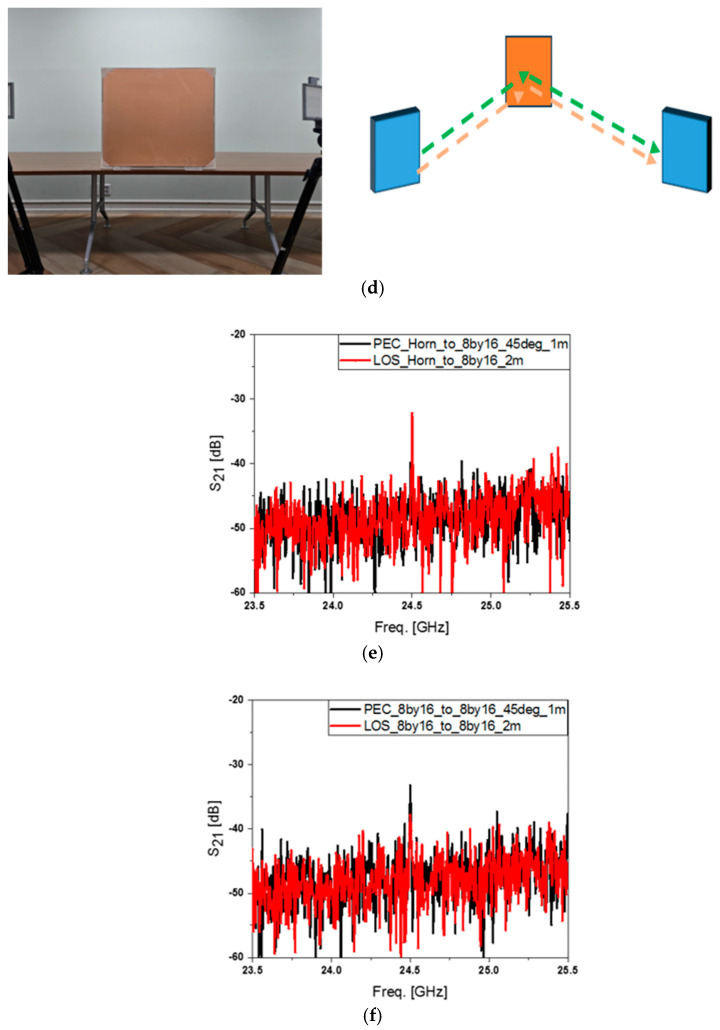
The preliminary tests on surface sensing. (**a**) LoS of the horn antenna to the 8-by-16 array antenna. (**b**) LoS of two 8-by-16 array antennas. (**c**) A large reflector between the horn and the 8-by-16 array antenna. (**d**) A large reflector between the two 8-by-16 array antennas. (**e**) compares (**a**) and (**c**) LoS values and (**f**) compares (**b**) and (**d**).

**Figure A2:**
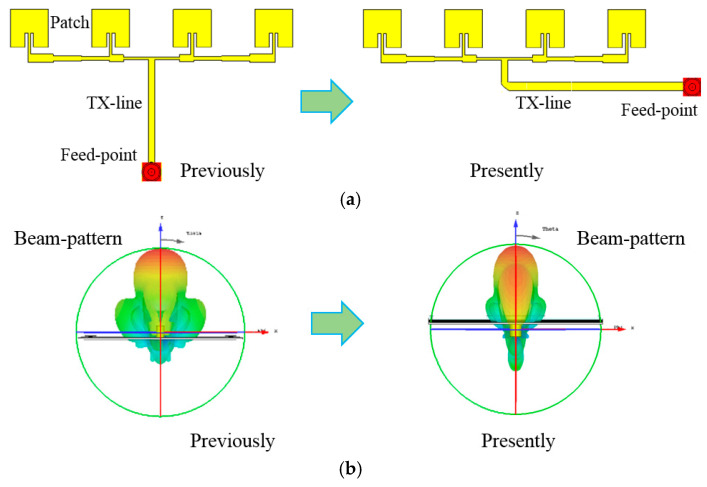
Comparison of the common and proposed array antennas for the wireless communication link. (**a**) Geometries with different feeding schemes. (**b**) Radiated wave patterns of the common and proposed array antennas expressed in from red (strongest) through green (middle) to blue (weakest).

**Figure A3:**
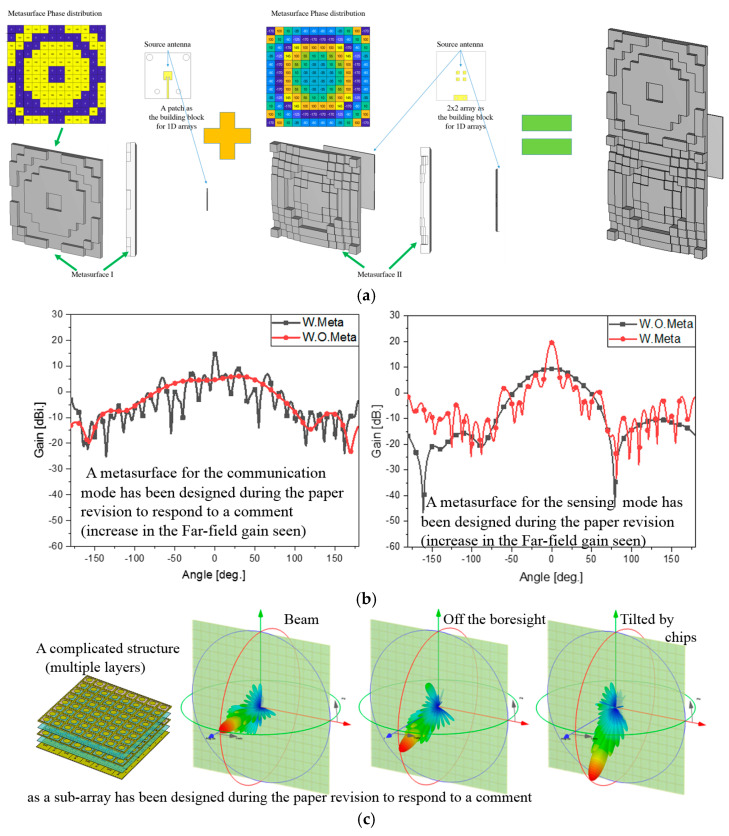
Different antennas having been designed in this lab as alternative approaches. (**a**) Metamaterial lenses for two frequencies as a new approach. (**b**) Beam directivity is improved by the use of metamaterial lenses. (**c**) Sub-array antenna requiring multiple layers to tilt the beam, when fed by the phase-set from beamformer chips expressed in from red (strongest) through green (middle) to blue (weakest).

**Figure A4:**
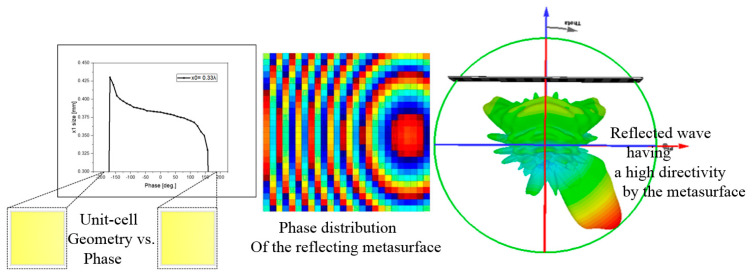
Detailed aspects of the reflecting metasurface used in the test. Unit cell and its phase function vs. geometry and the phase distribution on the surface and the reflected beam expressed in from red (strongest) through green (middle) to blue (weakest).

## Data Availability

The original contributions presented in the study are included in the article, further inquiries can be directed to the corresponding author.

## Appendix A

Before handling small-sized surfaces, such as DUTs, experimental configurations were tested and investigated with a large-sized reflecting surface, as is performed for RF calibration.

Figure A1a has a horn antenna and an 8-by-16 array antenna on the line-of-sight (LoS), whose distance is 2 m. Two 8-by-16 array antennas see each other directly in Figure A1b. The TX side and RX side are connected to the signal generator and spectrum analyzer, respectively. The signal generator is used in the preliminary tests because its input power is adjustable by up to 15 dBm greater than that of the VNA. For the sake of surface sensing, the proposed scheme deals with relatively small reflecting surfaces, whereas the setup here adopts a relatively large reflecting geometry whose size is 1 m by 1 m. This is placed leaning against the wall to generate the reflection of the signal from the TX antenna to the RX antenna. The angles of incidence and reflection are set at 45 degrees and the travelling distance from the TX to RX antenna is 2 m, as in Figure A1c,d. The received signal strength taken in the spectrum analyzer for the four cases is presented in Figure A1e,f. For the setup using the horn and the array antenna, the signal strength of the LoS case overlaps that of the reflection by the large metal plane, as shown in Figure A1e. The value is −35 dBm. A similar effect is observed in Figure A1f, where the reflected wave caused by the large metal scatterer has almost the same received power level as that of the LoS between the two array antennas. Based on these experiments, the surface-sensing antenna integrated with the 5G antenna is adopted to sense the smaller-sized metal- and metamaterial-surfaced structures.

Back to the origin of the proposed idea, more explanations need to be given regarding the basics of the mm-wave antenna, apart from Section 2, and concerning the reflecting metasurface chosen as an object for RF sensing. Firstly, for the communication link made at 28 GHz, a 1D array antenna is designed as in Figure 3a and in the following. It has a feed-line different from the conventional one to make the RF signals stronger.

One of the best ways to make RF signals that emanate from the antenna strong is to increment the directivity of the radiated beam. The shape of the feed-line matters for the 1D array antenna, unlike with larger apertures or 2D arrays. The main feeding TX-line is parallel to the orientation of the patches, as this is the commonly used method. Contrarily, the feed-line is bent at a right angle, and most of the TX-line is proposed to be orthogonal to the orientation of the patches, as shown in Figure A2a. It aims to suppress the interruption of the waves from the feeding TX-line by twisting the polarization. Figure A2b shows the changed beam-width or, in other words, the improvement in directivity, which renders the gain to an increase from 5 to 8.dBi. This provides positive effects on the mm-wave communication. Secondly, researchers might choose to use shared aperture metamaterials and sub-arrays instead of the proposed antenna structure, which may seem simple. When surveying technical articles, like [21,22], a number of them seen to give intriguing functions for metamaterial antennas or sub-arrays for mm-wave frequencies. In line with their proposals, a variety of mm-wave metamaterial antennas have been designed in this lab, but due to a tendency for the volume of each of the structures with accessories becoming large, an inexpensive and effective one was suggested instead.

As a reviewer of this paper, noted shared aperture antennas or sub-arrays should be tried instead of our structure, and they implied that there are novel antennas better than ours, so we have spared time to design antennas different from the proposed structure. Shared apertures introduced in [21,22] might have been tried at the beginning. R. Xiao et al.’s antenna works for the Ku band and W band. W. Niu et al. obtained far-fields from 3.4 to 3.8 GHz for a high-band and from 0.69 to 0.96 GHz for a low-band. The two bands of the dual-band shared aperture are very far away from each other, enabling big and small elements to be alternately mounted, whereas the two bands of our antenna module are too close to adopt the shared aperture. When it comes to a novel structure, we can solve this problem by proposing the metamaterial lenses shown in Figure A3a. A metamaterial lens for a 28 GHz single patch can be combined with the one for a 24 GHz two-by-two patch array. Each of the lenses comprises the unit cells of heights on PPS as the dielectric superstrate equivalent to the phases distributed as colors marked on the grid. They increase the antenna gains of the 28 GHz and 24 GHz primary antennas by more than 11 dB, as observed in Figure A3b. Nevertheless, the reason that this new antenna is not adopted for the work of this paper is the structure becomes much bigger than the proposed antenna, resulting from the enlarged area and the gap between the metamaterial lens and its source antenna. Additionally, it requires jigs to hold the lenses, which cost more to realize. Sub-arrays have also been designed in this lab, though this is not the main focus of this paper. Figure A3c shows a 3D view of the geometry and the beam-patterns for steering at 24 GHz. Because this system is made up of multiple layers with horizontal elements interconnected to vertical elements, it is costly to implement. One of the reasons to use sub-arrays is to steer the beam. This necessitates the use of beamformer chips, resulting in an increased cost for connecting, debugging, and controlling. This is not suitable for developing lighter and less bulky antennas, many which will be deployed on automobiles, cube satellites, and as access points in mmW networks. When our antenna is applied to the idle nodes selected to work as relays in the cooperative network, the QoS will be enhanced by reducing the blockage error and unnecessary power consumption, as in [23]. Lastly, Figure 5 explains RS01 in more detail. Actually, there are many candidates for a reflecting surface other than the metal plane. It does not have to be a metasurface. Any arbitrary pattern is taken, because the objective of the experiments is to see whether differences occur in S_21_ and the transmission ratio as the object changes in the sensing path. The metasurface is discussed in detail in the following figures.

The phase distribution on the surface is needed to assure the user of a high directivity at the desired angle of reflection, as in Figure A4, and this is rooted in the unit cell given on the left of the picture. The phase of the unit cell is the function of the size of the metal part (in yellow) on the 4350 B substrate, and the size is determined to meet the phase required by the value given from the phase distribution in the middle. The reflection from all the cells will be summed to tilt the incident wave to 45 degrees as the angle of reflection here. This direction is aligned with the path from the plane to the receiver.
